# Peri‐ictal respiratory dysfunction: Expanding the association between mTOR pathway disorders and ictal central apnea

**DOI:** 10.1111/epi.18646

**Published:** 2025-09-19

**Authors:** Margherita Burani, Giada Giovannini, Niccolò Orlandi, Matteo Pugnaghi, Leonardo Affronte, Mara Malerba, Lisa Taruffi, Laura Madrassi, Simona Scolastico, Alice Ballerini, Anna Elisabetta Vaudano, Irene Florindo, Enrico Ambrosini, Elisa Micalizzi, Gian Marco Duma, Elisa Osanni, Alberto Danieli, Fabiana Mambretti, Paolo Bonanni, Stefano Meletti

**Affiliations:** ^1^ Department of Biomedical, Metabolic and Neural Sciences University of Modena and Reggio Emilia Modena Italy; ^2^ Neurophysiology Unit and Epilepsy Center, Neuroscience Department Modena Azienda Ospedaliera‐Universitaria Modena Italy; ^3^ Neurology Unit, Department of Medicine and Surgery University Hospital of Parma Parma Italy; ^4^ Medical Genetics Unit University Hospital of Parma Parma Italy; ^5^ Neurophysiology Unit, Department of Neuroscience IRCCS San Martino Hospital Genoa Italy; ^6^ Epilepsy and Clinical Neurophysiology Unit Scientific Institute IRCCS E. Medea Conegliano Italy; ^7^ Laboratory of Medical Genetics Scientific Institute IRCCS E. Medea Bosisio Parini Italy

**Keywords:** *DEPDC5*, epilepsy, ictal central apnea, mTOR, *NPRL3*, SUDEP

## Abstract

Among the etiologies of focal epilepsy, mutations of the GATOR1 complex genes—comprising *NPRL3*, *NPRL2*, and *DEPDC5*—are known to result in overactivation of mTORC1. A recent study highlighted an association between ictal and postictal central apnea (ICA) and pathogenic variants of *DEPDC5*. Here, we analyzed data from 134 patients across two independent cohorts diagnosed with focal epilepsy who underwent video‐electroencephalographic long‐term monitoring (VLTM) with cardiorespiratory polygraphy. Genetic testing results done for clinical–diagnostic purposes were reviewed in patients with epilepsy of unknown etiology and patients with magnetic resonance imaging (MRI)‐defined/suspected focal cortical dysplasia (FCD). In 46 patients, we recorded at least one seizure associated with ICA. Genetic testing was performed in 21 of 22 MRI‐negative patients with ICA, revealing variants in mTOR pathway genes in 10 cases (48%), including *DEPDC5* (*n* = 6), *NPRL3* (*n* = 3), and *MTOR* (*n* = 1). Regarding MRI‐positive patients with ICA (*n* = 24), an acquired lesional etiology was found in 11. Of 13 patients with MRI‐defined FCD, genetic testing was carried out in seven, all of whom had negative results. Moreover, no pathogenic variants were detected in the 14‐MRI negative patients without ICA. Our findings confirm that variants in mTOR pathway genes (not only in *DEPDC5*) are present in patients with ICA and underline the potential risk of sudden unexpected death in epilepsy. These results also highlight the importance of performing respiratory polygraphy during VLTM to document ictal apnea.

## 1 INTRODUCTION

In focal epilepsy due to genetic etiology,^1^ growing attention has been directed to genes involved in the mTOR signaling pathway, which triggers many biological processes including cell growth, proliferation, and apoptosis, primarily through regulation of the cell cycle.^2^ mTOR is ubiquitously expressed, with high levels especially in the brain. It operates through two complexes: complex 1 (mTORC1) and complex 2 (mTORC2). The mTORC1 pathway regulates synaptic transmission and plasticity, neural network activity, neurogenesis, and neuronal morphology. Dysregulation of the pathway is implicated in the development of both genetic and acquired epilepsies. The mTORC1 pathway is upregulated in several neurological conditions associated with malformations of cortical development and drug‐resistant seizures. These conditions include focal cortical dysplasia (FCD), hemimegaloencephaly, and tuberous sclerosis complex.^3^

Germline mutations have been found in genes encoding the components of the GATOR1 complex (*DEPDC5, NPRL2, NPRL3*), a repressor of mTORC1. These mutations are increasingly recognized to cause a wide spectrum of focal epilepsy syndromes, with or without visually detected cortical structural abnormalities on magnetic resonance imaging (MRI). Although the role of the GATOR2 complex genes in focal epilepsy and/or FCD cannot be definitively excluded, the frequency of mutations at least seems to be low.^2^

Recent findings in animal models suggest a role of *DEPDC5* pathogenic variants in peri‐ictal respiratory dysfunction leading to terminal apnea and death.^4^ Our group has recently investigated respiratory alterations during seizures in a cohort of patients with focal epilepsy of unknown etiology (MRI negative), describing five patients with ictal central apnea (ICA) and pathogenic *DEPDC5* variants.^5^

A mechanistic understanding of epilepsy related to GATOR1 variants and the role of ictal respiratory dysfunction is extremely relevant and can guide future studies to more accurate diagnosis and treatments. The application of cardiorespiratory monitoring and genetic testing in these patients may contribute to reducing the risk of sudden unexplained death in epilepsy (SUDEP) and to improving genetic diagnostic counseling for families. This knowledge could also allow a precision medicine approach with drug targets based on the inhibition of the mTORC1 pathway.

Here, we expand the occurrence of ictal respiratory alterations in patients harboring variants in different mTORC1 pathway genes beyond *DEPDC5*.

## 2 MATERIALS AND METHODS

A cohort of 134 consecutive patients with focal epilepsy was evaluated at two epilepsy centers (Modena Academic Hospital and IRCCS E. Medea, Conegliano, Italy) from April 2020 to January 2025 to characterize ictal respiratory alterations. All the patients were admitted to the epilepsy monitoring unit and underwent: (1) long‐term video‐electroencephalographic (EEG) monitoring (LTVM), including overnight polysomnography at least once; (2) a high‐field (3 T) brain MRI study with a dedicated epilepsy protocol; and (3) cerebrospinal fluid analysis when clinically indicated (suspect of autoimmune encephalitis, infectious, inflammatory causes).

Each patient underwent LTVM with a 10–20 EEG system integrated with a standard precordial single‐channel ECG, pulse oximetry for SpO_2_ measurement, and a thoracoabdominal belt for respiratory inductance plethysmography. Obstructive sleep apnea was excluded based on clinical history and at least one overnight study performed without nasal airflow. According to published criteria,^6^, ^7^, ^8^, ^9^, ^10^ ICA was defined as a respiratory arrest lasting ≥5 s, visible on the pneumographic channel, and preceded and followed by stable breathing for at least 5 s. Postictal apnea was identified as a respiratory arrest starting within 5 s after the termination of ictal discharge or as apnea persisting after seizure termination.^11^ Data collected included apnea duration, hypoxemia (duration, nadir, and degree of oxygen desaturation), apnea awareness, and heart rate changes.

Hypoxemia was defined as a drop in SpO_2_ value to <95% and classified as mild (90%–94%), moderate (75%–89%), or severe (<75%).^6^, ^7^, ^8^, ^9^, ^10^ Tachycardia and bradycardia were defined as heart rate of >100 beats per minute and <60 beats per minute, or a >20% deviation from baseline, respectively.^9^

In this cohort of patients with focal epilepsy, a genetic analysis for clinical–diagnostic purposes was offered to patients without a definite etiology (i.e., epilepsy of unknown etiology) at the end of the diagnostic workup or to patients with MRI‐defined/suspected FCD. At both centers, DNA was extracted from blood samples and analyzed by next generation sequencing (NGS) with custom panels covering 196–216 genes associated with epilepsy, including genes known to cause epilepsies related to alterations in the mTORC1 pathway.^12^, ^13^ In one patient (PR01) a single nucleotide polymorphism array analysis (GenetiSure Dx Postnatal Assays, Agilent) was needed for a complete molecular diagnosis.

### 2.1 Standard protocol approvals, registrations, and patient consents

The study was approved by the local ethical committees (Area Vasta Emilia Nord N.322/15 and 679/2022/SPER/UNIMO; CET Area Nord Veneto N.22494).

Patients gave written informed consent for the use of their clinical records in this study. The study was conducted in accordance with the World Medical Association Declaration of Helsinki.

## 3 RESULTS

Polygraphic recordings showed the occurrence of seizures with ICA in 46 of 134 patients (34.3%). At the end of the diagnostic workup, 22 of 46 patients were classified as epilepsy of unknown etiology and were offered NGS genetic testing, which was finally performed in 21 patients.

**FIGURE 1:**
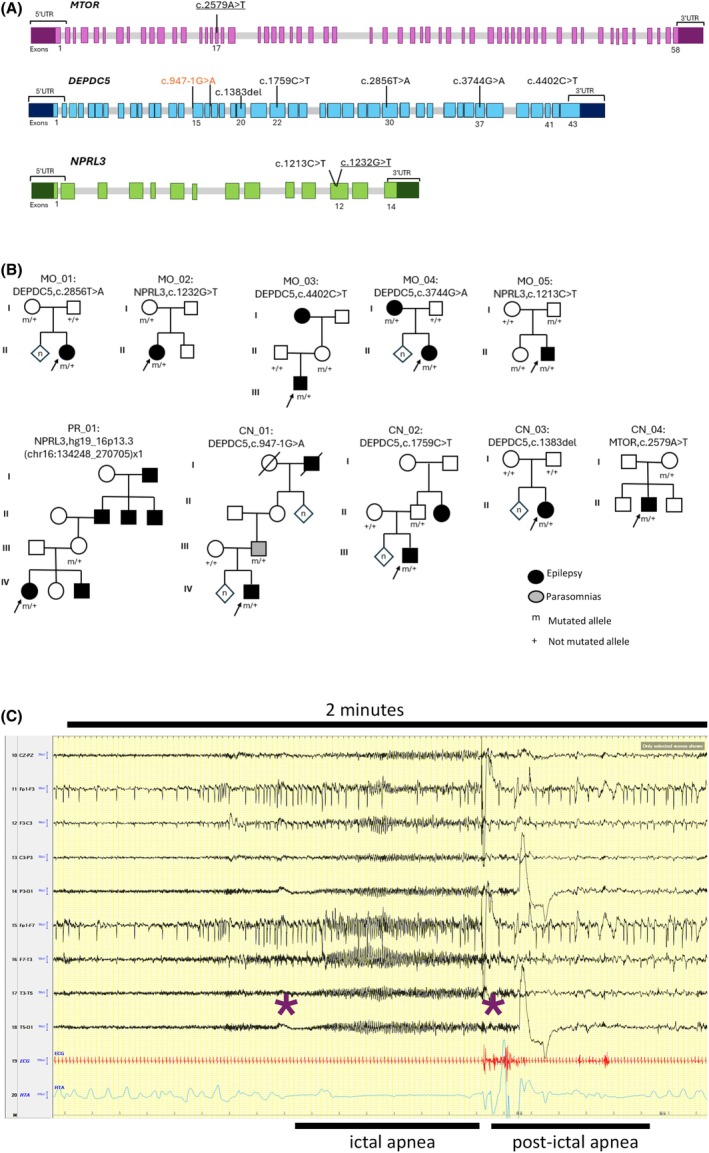
(A) Schematic structures of mTORC1 pathway genes with the position of point variants found in our patients. Variants indicated in black are nonsense or frameshift mutations, variants in orange are predicted to alter mRNA splicing, and missense variants are underlined. The microdeletion detected in Patient PR_01, encompassing the entire *NPRL3* gene, is not represented. All mutations are classified as pathogenic or likely pathogenic according to the American College of Medical Genetics and Genomics criteria, except c.1232G>T in *NPRL3* and c.2579A>T in *MTOR*, both classified as variants of uncertain significance. (B) Pedigrees of families with the proband indicated by an arrow. Individuals with a mutation are indicated by m/+, and individuals negative for the mutation are indicated by +/+. Unknown family member are identified by n. (C) Two minutes of long‐term polysomnography showing a left onset seizure with frontotemporal rhythmic activity evolving to 3–4‐Hz spike and waves lasting approximately 40 s, followed by slow activity for approximately 30 min. The seizure onset and offset are marked with purple asterisks. The respiratory trace shows a breath stop of approximately 30 s followed by respiratory irregularity. The patient was sitting on her bed and was using the phone. Clinically, she stopped and presented tonic head right deviation. Called by the technician, she did not answer or carry out orders. The patient later reported no awareness of the episode.

In these patients (mean age = 29.1 years, age range = 7–59), 10 variants in genes coding for proteins that are involved in the mTORC1 signaling pathway have emerged, including *DEPDC5* (*n* = 6), *NPRL3* (*n* = 3), and *MTOR* (*n* = 1; Figure 1A,B; Table S1). Patients MO_01, MO_04, and CN_01–03 harboring *DEPDC5* pathogenic variants were previously reported.^5^ No other pathogenic variants in epilepsy genes were found.

Table 1 shows the patients' clinical features. Briefly, patients' mean age at epilepsy onset was 16.7 years (SD = 13.1), a family history of epilepsy was reported in 30.4% of individuals, and 76.1% had drug‐resistant epilepsy. Psychiatric comorbidities were reported in two individuals with a variant in *DEPDC5*, with psychotic events. No one in this cohort had intellectual disability.

**TABLE 1:** Clinical features of patients with ictal central apnea and mTORC1 pathway pathogenic variants.

Characteristic	Patients, *n* = 10
Mean age, years (minimum–maximum)	29.1 (7–59)
Female, *n* (%)	5 (50%)
Duration of epilepsy at study time, years, mean (median)	20.5 (16.5)
Age at epilepsy onset, years, mean (median)	8.6 (7.5)
Family history of epilepsy, *n* (%)	5 (50%)
History of febrile seizures, *n* (%)	1 (10%)
Drug‐resistant epilepsy, *n* (%)	8 (80%)
Epilepsy type, *n* (%)	
Focal	10 (100%)
TLE	7 (70%)
FLE	2 (20%)
Insulo‐opercular epilepsy	1 (10%)
Genetic etiology, *n* (%)	10 (100%)
*DEPDC5*	6 (60%)
*NPRL3*	3 (30%)
*MTOR*	1 (10%)
Intellectual disability, *n* (%)	0
Psychiatric comorbidity, *n* (%)	
Psychosis	2

Abbreviations: FLE, frontal lobe epilepsy; TLE, temporal lobe epilepsy.

Regarding the 24 MRI‐positive patients with ictal apnea, all were considered to have temporal lobe epilepsy based on electroclinical findings. The following etiologies were observed: FCD (*n* = 13), low‐grade epilepsy‐associated tumor (*n* = 2), low‐grade glioma (*n* = 2), encephalocele (*n* = 1), hippocampal sclerosis (*n* = 3), postsurgery gliotic scar (*n* = 2), and postherpes encephalitis (*n* = 1). In this group of patients, NGS epilepsy panel was offered to patients with FCD and was finally obtained in seven patients, all negative for pathogenic variants (Table S2).

Finally, NGS testing was also performed in 14 patients with focal epilepsy of unknown etiology without ICA; none of these subjects showed a pathogenic variant related to epilepsy genes.

### 3.1 Peri‐ictal respiratory findings and ictal semiology

Patients with mTORC1 pathway mutation (*n* = 10) showed seizure‐related respiratory alterations in each recorded seizure with varying degrees of severity (Figure 1C). None of the patients had a history of snoring or sleep apnea, and overnight sleep polygraphy did not show obstructive apnea in any patient.

During LTVM, we recorded 31 seizures with ICA, 23 (74.2%) occurring during sleep: 19 during N2, four during N3, and none in N1 or rapid eye movement sleep. All the recorded seizures had focal onset, starting from temporal (*n* = 7) or frontal (*n* = 3) regions. The seizures were characterized by brief EEG arousal, without any visible movement. In a few cases, the video showed brief clinical awakenings, with early falling back to sleep. Patients tended to change their position, rotate, return to stillness, and then fall asleep again. No episodes with tonic–clonic evolution or rhythmic/hyperkinetic movements were recorded. In Table S3, we summarize the characteristics of each event; the average duration of the apnea was 35.32 s (maximum duration = 140 s). In 35.5% of cases, we observed oxygen desaturation, up to a nadir of 71% SpO_2_. In all events, the ictal apnea was associated with tachycardia. All patients appeared unaware and denied any feeling of stopping breathing or respiratory distress when asked.

Postictal breathing alterations were recorded in four of 10 patients, and three of them (75%) carried *NPRL3* variants, meaning that three of three patients harboring a pathogenic variant in the *NPRL3* gene exhibited apnea persisting in the postictal period. Postictal apnea was never observed without concomitant ICA. Seizures with postictal apnea (*n* = 8) showed a longer peri‐ictal apnea time (mean = 64 s vs. 25 s, *p* = .010) and a longer desaturation (35 s vs. 9 s, *p* = .005) compared with seizure with self‐limiting ICA (*n* = 23), despite similar SpO_2_ nadir being recorded (71% vs. 75%). The mean duration of the ictal discharge was similar in the two groups (47 s vs. 52 s).

## 4 DISCUSSION

We have documented 10 patients with focal epilepsy carrying pathogenetic variants (8/10) or variants of uncertain significance (2/10) in the mTORC1 pathway genes (*DEPDC5, NPRL3*, and *MTOR*) who developed central apnea in 100% of their seizures, for a total of 31 seizures with respiratory alterations. We therefore expand previous observations of an association between peri‐ictal central apnea and *DEPDC5* to other genes involved in the same pathway. Although the patient cohort is limited, our data support an association between peri‐ictal respiratory changes and the mTORC1 signaling pathway. Therefore, given the link between mTOR‐related epilepsy and an increased risk of SUDEP,^9^, ^14^ cardiorespiratory monitoring holds significant relevance in clinical settings.

### 4.1 Sleep‐related “hypomotor” phenotype

In recent years, due to growing recognition of the role of GATOR1 genes in the pathogenesis of focal epilepsies, researchers have aimed to delineate a more precise phenotypic spectrum.^13^ The “GATOR1 phenotype” has been commonly described as sleep‐related hypermotor epilepsy (SHE),^15^ leading clinicians to routinely investigate potential genetic etiologies of sleep‐related seizures with vigorous hyperkinetic movements and/or tonic or dystonic asymmetric posturing, with or without impaired awareness.^16^

In our cohort of patients carrying GATOR1 complex variants, seizure semiology includes arousal with breathing arrest, tachycardia, slow/nonjerky positional adjustments, and in some cases, continuation of sleep. This clinical presentation may be more consistent with a “hypomotor” phenotype. Regarding tachycardia, which was a constant finding in this cohort, we believe it represents a peri‐ictal autonomic response not specifically related to the effect of mTOR pathway genes on cardiac function.

Of note, most of our patients (6/10) exhibited focal epilepsy with temporal lobe onset, in contrast to the 70% frontal lobe onset typically observed in SHE.^17^ Recognizing these features may improve the use of genetic tests, highlighting the need to investigate GATOR1 variants even in sleep‐related focal epilepsies with subtle or nonmotor seizure semiology.

### 4.2 Amygdala, ICA, and mTOR

Why should peri‐ictal respiratory changes be associated with mutations in mTOR pathway genes? To date, we do not have a precise answer. Previous studies reported volumetric increase of the amygdala and overactivity markers of the mTOR pathway in amygdala tissue in patients with ICA.^7^, ^18^ Moreover, a close relationship between ICA and amygdala involvement was demonstrated by intracranial EEG ictal recordings and intracerebral electrical stimulation studies.^19^, ^20^ Of note, amygdala electrical stimulation has been reported to increase blood oxygen level‐dependent functional MRI signal in the brainstem in man, and very recently brainstem hyperperfusion (ictal single photon emission computed tomography) has been observed during ictal apnea.^21^, ^22^ Finally, we disclosed enlarged amygdala in patients with ictal/postictal breathing alterations compared to patients without seizure‐related apnea and healthy controls, supporting the role of amygdala in the generation of seizure‐related respiratory alterations.^7^, ^11^

### 4.3 Study limitations and future perspectives

This study was not a prospective investigation aimed at assessing the frequency of pathogenic mutations in patients with epilepsy and ictal respiratory changes. Our study does not intend to claim a causal specificity of mTOR pathogenic variants in the genesis of ictal respiratory alterations. Instead, we want to bring light on the association between mTOR genes and peri‐ictal respiratory alterations. Our research is built upon earlier studies focused on evaluating the incidence and characteristics of ictal/postictal respiratory alterations in patients admitted to epilepsy monitoring units. Consequently, our findings should be interpreted cautiously and viewed as preliminary, warranting further exploration in larger patient populations. Nevertheless, our results could lead to new studies aimed at deepening our understanding of epilepsy associated with GATOR1 variants and its clinical features, to inform clinical decision‐making, personalize treatments, and support genetic counseling. Of note, we showed a high frequency of germline mTOR pathogenic variants in our MRI‐negative patients with ICA (approximately 50%), but it should also be underscored that approximately 50% of MRI‐positive patients with ICA had an MRI‐visible FCD. Considering that FCD can result from somatic mutation in mTOR pathway genes,^23^ future studies should also evaluate genetic testing on surgical specimens in such patients, an analysis that was not possible in our cohort.

## AUTHOR CONTRIBUTIONS

**Margherita Burani:** Conceptualization (equal); data curation (lead); formal analysis (lead); writing—original draft (equal); writing—review and editing (equal). **Stefano Meletti:** Conceptualization (lead); data curation (lead); formal analysis (lead); writing—original draft (equal); writing—review and editing (equal). **Paolo Bonanni:** Supervision (lead); conceptualization (equal); visualization (equal); writing—original draft (equal); writing—review and editing (equal). **Gian Marco Duma:** Data curation (lead); formal analysis (lead); writing—original draft (equal); writing—review and editing (equal). **Fabiana Mambretti:** Data curation (lead); formal analysis (lead); writing—original draft (equal); writing—review and editing (equal). **Giada Giovannini, Niccolò Orlandi, Leonardo Affronte, Mara Malerba, Matteo Pugnaghi, Lisa Taruffi, Laura Madrassi, Simona Scolastico, Alice Ballerini, Anna Elisabetta Vaudano, Irene Florindo, Enrico Ambrosini, Elisa Osanni, Alberto Danieli, Elisa Micalizzi:** Data curation (equal); writing—review and editing (equal).

## FUNDING INFORMATION

This work was supported by funds obtained from the Italian Ministry of Health, grant Ricerca Corrente to IRCCS Medea (F.M.).

## CONFLICT OF INTEREST STATEMENT

S.M. has received research grant support from the Ministry of Health (MOH); and has received personal compensation as a scientific advisory board member for UCB, Jazz Pharmaceuticals, and Eisai. A.E.V. has received speaker's or consultancy fees from Angelini. P.B. has received research grant support from the MOH; and has received speaker's, consultancy, or scientific advisory board member fees from Angelini, LivaNova, Eisai, UCB, Jazz Pharmaceuticals, and Biocodex. None of the other authors has any conflict of interest to disclose.

## ETHICS STATEMENT

The study was approved by the local ethical committee (NET‐2013‐02355313 N.155/14; N. 238/23). Patients gave written informed consent for the use of their clinical records in this study. The study was conducted in accordance with the World Medical Association Declaration of Helsinki. Data are reported according to the STROBE checklist for clinical studies. We confirm that we have read the Journal's position on issues involved in ethical publication and affirm that this report is consistent with those guidelines.

## Supporting information


Table S1.

## Data Availability

Data will be shared upon reasonable request to the corresponding author.

## References

[1] Guerrini R , Balestrini S , Wirrell EC , Walker MC . Monogenic epilepsies: disease mechanisms, clinical phenotypes, and targeted therapies. Neurology. 2021;97(17):817–831. 10.1212/WNL.0000000000012744 34493617 PMC10336826

[2] Baulac S . mTOR signaling pathway genes in focal epilepsies. Prog Brain Res. 2016;226:61–79. 10.1016/bs.pbr.2016.04.013 27323939

[3] Crino PB . mTOR: a pathogenic signaling pathway in developmental brain malformations. Trends Mol Med. 2011;17(12):734–742. 10.1016/j.molmed.2011.07.008 21890410

[4] Kao HY , Yao Y , Yang T , Ziobro J , Zylinski M , Mir MY , et al. Sudden unexpected death in epilepsy and respiratory defects in a mouse model of DEPDC5‐related epilepsy. Ann Neurol. 2023;94(5):812–824. 37606181 10.1002/ana.26773 PMC10592102

[5] Meletti S , Duma GM , Burani M , Danieli A , Giovannini G , Osanni E , et al. Ictal and postictal central apnea in DEPDC5‐related epilepsy. Neurol Genet. 2024;10(5):e200183. 10.1212/NXG.0000000000200183 39376210 PMC11458130

[6] Micalizzi E , Vaudano AE , Ballerini A , Talami F , Giovannini G , Turchi G , et al. Ictal apnea: a prospective monocentric study in patients with epilepsy. Eur J Neurol. 2022;29(12):3701–3710. 36057450 10.1111/ene.15547 PMC9826458

[7] Micalizzi E , Ballerini A , Giovannini G , Cioclu MC , Scolastico S , Pugnaghi M , et al. The role of the amygdala in ictal central apnea: insights from brain MRI morphometry. Ann Clin Transl Neurol. 2024;11(1):121–132. 37936526 10.1002/acn3.51938 PMC10791031

[8] Lacuey N , Zonjy B , Hampson JP , Rani MRS , Zaremba A , Sainju RK , et al. The incidence and significance of periictal apnea in epileptic seizures. Epilepsia. 2018;59(3):573–582. 29336036 10.1111/epi.14006 PMC6103445

[9] Vilella L , Lacuey N , Hampson JP , Rani MRS , Loparo K , Sainju RK , et al. Incidence, recurrence, and risk factors for Peri‐ictal central apnea and sudden unexpected death in epilepsy. Front Neurol. 2019;10:166. 30890997 10.3389/fneur.2019.00166 PMC6413671

[10] Bateman LM , Li CS , Seyal M . Ictal hypoxemia in localization‐related epilepsy: analysis of incidence, severity and risk factors. Brain. 2008;131(12):3239–3245. 18952672 10.1093/brain/awn277 PMC2639205

[11] Meletti S , Burani M , Ballerini A , Giovannini G , Micalizzi E , Orlandi N , et al. Persistent postictal central apnea in focal seizures: incidence, features, and imaging findings. Neurology. 2025;105(4):e213856. 40694793 10.1212/WNL.0000000000213856 PMC12288844

[12] Baulac S , Baldassari S . DEPDC5‐related epilepsy. In: Adam MP , Feldman J , Mirzaa GM , Pagon RA , Wallace SE , Bean LJH , et al., editors. GeneReviews®. Seattle (WA): University of Washington, Seattle; 2016. p. 1993–2024. 27683934

[13] Baldassari S , Picard F , Verbeek NE , van Kempen M , Brilstra EH , Lesca G , et al. The landscape of epilepsy‐related GATOR1 variants. Genet Med. 2019;21(2):398–408. 30093711 10.1038/s41436-018-0060-2 PMC6292495

[14] Schuele SU , Afshari M , Afshari ZS , Macken MP , Asconape J , Wolfe L , et al. Ictal central apnea as a predictor for sudden unexpected death in epilepsy. Epilepsy Behav. 2011;22(2):401–403. 10.1016/j.yebeh.2011.06.036 21816679

[15] Licchetta L , Pippucci T , Baldassari S , Minardi R , Provini F , Mostacci B , et al. Sleep‐related hypermotor epilepsy (SHE): contribution of known genes in 103 patients. Seizure. 2020;74:60–64. 10.1016/j.seizure.2019.11.009 31835056

[16] Tinuper P , Bisulli F , Cross JH , Hesdorffer D , Kahane P , Nobili L , et al. Definition and diagnostic criteria of sleep‐related hypermotor epilepsy. Neurology. 2016;86(19):1834–1842. 10.1212/WNL.0000000000002666 27164717 PMC4862248

[17] Proserpio P , Cossu M , Francione S , Tassi L , Mai R , Didato G , et al. Insular‐opercular seizures manifesting with sleep‐related paroxysmal motor behaviors: a stereo‐EEG study. Epilepsia. 2011;52(10):1781–1791. 10.1111/j.1528-1167.2011.03254.x 21883183

[18] Lam HW , Patodia S , Zeicu C , Lim YM , Mrzyglod A , Scott C , et al. Quantitative cellular pathology of the amygdala in temporal lobe epilepsy and correlation with magnetic resonance imaging volumetry, tissue microstructure, and sudden unexpected death in epilepsy risk factors. Epilepsia. 2024;65:2368–2385. 38837385 10.1111/epi.18033

[19] Dlouhy BJ , Gehlbach BK , Kreple CJ , Kawasaki H , Oya H , Buzza C , et al. Breathing inhibited when seizures spread to the amygdala and upon amygdala stimulation. J Neurosci. 2015;35(28):10281–10289. 26180203 10.1523/JNEUROSCI.0888-15.2015 PMC4502266

[20] Lacuey N , Talavera B , Magana‐Tellez O , Mancera‐Páez O , Hupp N , Luo X , et al. Ictal central apnea is predictive of mesial temporal seizure onset: an intracranial investigation. Ann Neurol. 2024;95(5):998–1008. 38400804 10.1002/ana.26888 PMC11061876

[21] Harmata GIS , Rhone AE , Kovach CK , Kumar S , Mowla MR , Sainju RK , et al. Failure to breathe persists without air hunger or alarm following amygdala seizures. JCI Insight. 2023;8(22):e172423. 37788112 10.1172/jci.insight.172423 PMC10721319

[22] Kokkinos V , VanHaerents SA , Nathan CL , Ghandour D , Avery RJ , Schuele SU . Pontine hyperperfusion associated with ictal central apnea. Epileptic Disord. 2025;27:668–673. 40407738 10.1002/epd2.70047

[23] Macdonald‐Laurs E , Leventer RJ . ILAE genetic literacy series: focal cortical dysplasia. Epileptic Disord. 2025;27:1–8. 39641771 10.1002/epd2.20308 PMC11829622

