# Supplementary material for: Peri‐ictal respiratory dysfunction: Expanding the association between mTOR pathway disorders and ictal central apnea
Source: Epilepsia. 2025 Sep 19;66(11):e248–55. doi: 10.1111/epi.18646 (PMC12661263; doi:10.1111/epi.18646)
Supplement: Supplementary file 1 — Table S1. [file EPI-66-e248-s001.docx]

**Supplementary Materials**

**“Peri-ictal respiratory dysfunctions: expanding the association between mTOR pathway disorders and ictal central apnea”**

Burani et al.

*Supplementary Table 1: Sequence variants of genes found in our patients*

| MO_01 | DEPDC5, NM_001242896.3:c.2856T>A, p.(Cys952Ter), het, mat |
| --- | --- |
| MO_02 | NPRL3, NM_001077350.3:c.1232G>T, p.(Cys411Phe), het, mat |
| MO_03 | DEPDC5, NM_001242896.3:c.4402C>T, p.(Arg1468*), het, mat |
| MO_04 | DEPDC5, NM_001242896.3:c.3744G>A, p.(Trp1248*), het, mat |
| MO_05 | NPRL3, NM_001077350.3:c.1213C>T, p.(Gln405*), het, pat |
| PR_01 | NPRL3, GRCh37/hg19_16p13.3(chr16:134248_270705)x1, het, mat |
| CN_01 | *DEPDC5*, NM_001242896.3:c.947-1G>A, het, pat |
| CN_02 | *DEPDC5*, NM_001242896.3:c.1759C>T, p.(Arg587*), het, pat |
| CN_03 | *DEPDC5*, NM_001242896.3:c.1383del, p.(Tyr462Metfs*55), het, *de novo* |
| CN_04 | *MTOR*, NM_004958.4: c.2579A>T, p.(Lys860Met), het, mat |

*Supplementary Table 2: Structural etiologies in MRI-positive patients with ictal central apnea. TLE=temporal lobe epilepsy; FCD=focal cortical dysplasia; HS=hippocampal sclerosis; LEAT=low grade epilepsy associated tumour; NP=not performed.*

| **Pt ID** | **Age** | **Gender** | **Age at epilepsy onset** | **Epilepsy/side** | **Etiology** | **NGS** | **Results from NGS** |
| --- | --- | --- | --- | --- | --- | --- | --- |
| MO_06 | 23 | F | 11 | TLE/right | FCD | Yes | negative |
| MO_07 | 49 | M | 2 | TLE/left | Low grade glioma | NP | - |
| MO_08 | 20 | M | 19 | TLE/left | Encephalocele | NP | - |
| MO_09 | 27 | M | 1 | TLE/left | HS | NP | - |
| MO_10 | 19 | M | 18 | TLE/left | Post-herpes encephalitis | NP | - |
| MO_11 | 23 | M | 21 | TLE/right | FCD | NP | - |
| MO_12 | 38 | M | 38 | TLE/left | FCD | NP | - |
| MO_13 | 42 | F | 31 | TLE/right | FCD | NP | - |
| MO_14 | 28 | F | 23 | TLE/left | Post-surgery gliotic scar | NP | - |
| MO_15 | 31 | M | 23 | TLE/right | FCD | NP | - |
| MO_16 | 20 | F | 20 | TLE/left | FCD | NP | - |
| MO_17 | 55 | F | 42 | TLE/left | HS | NP | - |
| MO_18 | 51 | M | 8 | TLE/right | Post-surgery gliotic scar | NP | - |
| MO_19 | 19 | F | 17 | TLE/left | FCD | Yes | negative |
| MO_20 | 32 | M | 29 | TLE/right | LEAT | NP | - |
| MO_21 | 41 | M | 40 | TLE/right | FCD | NP | - |
| MO_22 | 21 | M | 17 | TLE/right | LEAT | NP | - |
| MO_23 | 59 | F | 11 | TLE/left | Low grade glioma | NP | - |
| MO_24 | 38 | M | 15 | TLE/right | HS | NP | - |
| CN_05 | 48 | M | 29 | TLE/left | FCD | Yes | negative |
| CN_06 | 32 | F | 29 | TLE/right | FCD | Yes | negative |
| CN_07 | 43 | F | 1 | TLE/left | FCD | Yes | negative |
| CN_08 | 13 | F | 3 | TLE/right | FCD | Yes | negative |
| CN_09 | 21 | F | 1 | TLE/right | FCD | Yes | negative |

*Supplementary Table 3: Clinical features of seizures*

| **Patients with ICA and pathogenic variant of mTOR** | **Recorded seizures with ICA/**  **total seizures** | **Apnea duration**  **(mean, s)** | **Desat. Duration**  **(mean, s)** | **Desat. Nadir (%)** | **Sleep at seizure onset/**  **total seizures** | **Heart rate changes** | **Awareness of respiratory distress** |
| --- | --- | --- | --- | --- | --- | --- | --- |
| MO_01 | 4/4 | 40.3 | 53.0 | 71 | 4/4 | Tachycardia | No |
| MO_02 | 2/2 | 60.5 | 0.0 | 100 | 1/2 | Tachycardia | No |
| MO_03 | 6/6 | 17.3 | 2.5 | 91 | 6/6 | Tachycardia | No |
| MO_04 | 1/1 | 60.0 | 120 | 75 | 0/1 | - | No |
| MO_05 | 1/1 | 90.0 | 7.0 | 79 | 1/1 | Tachycardia | No |
| PR_01 | 1/1 | 140.0 | 62.0 | 72 | 1/1 | Tachycardia | No |
| CN_01 | 3/3 | 35.0 | NA | NA | 3/3 | - | No |
| CN_02 | 6/6 | 26.0 | NA | NA | 4/6 | Tachycardia | No |
| CN_03 | 1/1 | 25.0 | 26.0 | 92 | 0/1 | Tachycardia | No |
| CN_04 | 6/6 | 22.2 | 38.0 | 80 | 4/6 | Tachycardia | No |
